# Etanercept for patients with juvenile idiopathic arthritis: drug levels and influence of concomitant methotrexate: observational study

**DOI:** 10.1186/s12969-023-00801-2

**Published:** 2023-03-22

**Authors:** Tiina Levälampi, Johanna Kärki, Katariina Rebane, Paula Vähäsalo, Merja Malin, Liisa Kröger, Minna-Maija Grönlund, Maria Backström, Heini Pohjankoski, Hannu Kautiainen, Sakari Jokiranta, Kristiina Aalto

**Affiliations:** 1 Pediatric Research Center, New Children’s Hospital, Helsinki University Hospital, University of Helsinki, Helsinki, Finland; 2 Department of Pediatrics, Kanta-Häme Central Hospital, Hämeenlinna, Finland; 3 PEDEGO Research Unit, University of Oulu, Oulu, Finland; 4 Department of Pediatrics, Oulu University Hospital, Oulu, Finland; 5 Medical Research Center, Oulu University Hospital and University of Oulu, Oulu, Finland; 6 Department of Pediatrics, Tampere University Hospital, Tampere, Finland; 7 Department of Pediatrics, Kuopio University Hospital, Kuopio, Finland; 8 Department of Pediatrics, Turku University Hospital, Turku, Finland; 9 Department of Paediatrics, The Wellbeing Services County of Ostrobothnia, Vaasa, Finland; 10 Department of Pediatrics, Päijät-Häme Central Hospital, Lahti, Finland; 11 Primary Health Care Unit, Kuopio University Hospital, Kuopio, Finland; 12 Folkhälsan Research Center, Helsinki, Finland; 13 Department of Bacteriology and Immunology, University of Helsinki, MedicumHelsinki, Finland; 14 Tammer BioLab Ltd, Tampere, Finland

**Keywords:** Juvenile idiopathic arthritis, Etanercept, Drug concentration, Methotrexate

## Abstract

**Background:**

Etanercept (ETN) is widely used tumour necrosis factor (TNF) blocker in the treatment of juvenile idiopathic arthritis (JIA) when traditional synthetic disease modifying antirheumatic drug (sDMARD) therapy is not sufficient. There is limited information about the effects of methotrexate (MTX) on serum ETN concentration in children with JIA. We aimed to investigate whether ETN dose and concomitant MTX would effect ETN serum trough levels in JIA patients, and whether concomitant MTX have an influence on the clinical response in patients with JIA receiving ETN.

**Methods:**

In this study, we collected the medical record data of 180 JIA patients from eight Finnish pediatric rheumatological centres. All these patients were treated with ETN monotherapy or combination therapy with DMARD. To evaluate the ETN concentrations, blood samples of the patients were collected between injections right before the subsequent drug. Free ETN level was measured from serum.

**Results:**

Ninety-seven (54%) of the patients used concomitant MTX, and 83 (46%) received either ETN monotherapy or used sDMARDs other than MTX. A significant correlation was noted between ETN dose and drug level [*r* = 0.45 (95% CI: 0.33–0.56)]. The ETN dose and serum drug level were correlated (*p* = 0.030) in both subgroups – in MTX group [*r* = 0.35 (95% CI: 0.14–0.52)] and in non-MTX group [*r* = 0.54 (95% CI: 0.39–0.67)].

**Conclusion:**

In the present study, we found that concomitant MTX had no effect on serum ETN concentration or on clinical response. In addition, a significant correlation was detected between ETN dose and ETN concentration.

## Background

Juvenile idiopathic arthritis (JIA) is the most common chronic inflammatory arthritis in childhood [1]. In Finland, with a population of 5.5 million, including 922 000 children under 16 years of age, nearly 200 children are diagnosed as having JIA every year [2] according to International League of Associations for Rheumatology (ILAR) criteria [3]. Treatment of JIA is usually initiated with conventional, synthetic disease-modifying antirheumatic drugs (sDMARDs), typically methotrexate (MTX) [4]. More than half of patients with JIA benefit from this treatment and achieve remission. Nearly all of those who do not achieve remission with sDMARDS benefit from biological disease-modifying antirheumatic drug (bDMARD) treatment [5]. According to the American College of Rheumatology (ACR) recommendations [4, 6], when traditional sDMARD therapy is not sufficient for treating JIA, a tumour necrosis factor (TNF) blocker, including etanercept (ETN), can be added. The treatment of JIA in Finland is based on the ACR treatment recommendation and is in line with European care practices [7].

ETN, a dimeric fusion protein that comprises two extracellular portions of the TNF receptor 2 linked to the Fc portion of human immunoglobulin G1, was introduced nearly 30 years ago for treating rheumatoid arthritis (RA) [8] and for treating JIA [9]. In Finland, ETN has been used for JIA since February 2000, and the normal procedure is subcutaneous administration once a week, occasionally twice a week, according to the manufacturer’s instructions https://www.ema.europa.eu/en/medicines/human/EPAR/enbrel.

In a clinical trial simulation, subcutaneous ETN injections 0.8 mg/kg weekly and 0.4 mg/kg twice a week produced overlapping steady-state time-concentration profiles and corresponding clinical outcomes [10]. Similar results were reported by Langley et al. in their study of pediatric patients with psoriasis who received ETN 0.8 mg/kg weekly and pediatric patients with arthritis who received ETN 0.4 mg/kg twice weekly [11]. ETN can be administered alone or in combination, usually with MTX. Nevertheless, the effect of MTX on the serum trough concentration of ETN remains unclear [12].

In this study, we aimed to investigate whether concomitant MTX and ETN doses affect ETN serum trough levels in patients with JIA and whether concomitant MTX affects clinical response in patients with JIA receiving ETN.

## Methods

### Patients and methods

This observational retrospective study collected the medical record data of patients from eight Finnish pediatric rheumatological centres: five university hospitals and three within secondary referral hospitals. Patients who received ETN regularly from July 2014 to November 2017 for at least two weeks and were under 18 years old were included in the study. ETN treatment was accomplished by the decision of the pediatric rheumatologist. Serum samples for the concentration measurement were taken for clinical reasons, mainly to assist in dose adjustment to optimise the use of ETN and/or verification of individual compliance. Pharmacological treatment comprised ETN monotherapy or combination therapy, with or without sDMARD. All analysed patients were diagnosed as having JIA according to ILAR criteria [3].

The following patient data were collected: ETN initiation date, dose of the drug (mg/kg), body surface area using Mosteller modulation [13], concomitant sDMARDs, previous bDMARDs, height, weight, age, sex, diagnosis date, and type of JIA. Basic clinical disease information included the following: antinuclear antibody (ANA), human leucocyte antigen B27 (HLA-B27) result, rheumatoid factor (RF) level, cyclic citrulline peptide antibody (CCP-ab), patient’s global assessment of wellbeing (PaGA), measured on a visual analogue scale (VAS) from 0 to 100, physician's global assessment of disease activity (PhGA) on a VAS from 0 to 100, 10-joint juvenile disease activity score (JADAS10) at the time of ETN concentration measurements, and possible comorbidities (uveitis or inflammatory bowel disease).

To evaluate the ETN concentrations of the patients, blood samples were collected between injections right before the subsequent drug dose to enable trough concentration measurement. This was the first ETN concentration measurement. Free ETN level was measured from serum with the ELISA method by Sanquin Diagnostics (Amsterdam, the Netherlands) [14] subcontracted by the United Medix Laboratory (Helsinki, Finland). The target value for residual ETN concentrations was above 1.5 µg/mL [15–17].

### Ethics

This register-based study was performed by collecting clinical data from patient records. Therefore, according to Finnish legislation, no approval by an ethical committee or informed consent was required. Each hospital granted permission to collect the patient data.

### Statistics

Data are presented as means with standard deviation (SD), medians with interquartile range (IQR), or counts with percentages. Statistical significance between groups was evaluated using t test or chi-square test. When adjusting for confounding factors, an analysis of covariance or logistic regression model was applied. Relationship between ETN dose and concentration estimated according to the use of MTX by tuota moni ei mut intissäusing two separate univariate regression models. In the case of violation of the assumptions (e.g., non normality) for continuous variables, a bootstrap-type method or Monte Carlo p-values (small number of observations) for categorical variables were used. Correlation coefficients were calculated using the Spearman method, using Sidak-adjusted (multiplicity) probabilities. ETN dose adjusted (partial) correlation between dose of MTX and ETN serum trough level was calculated by the Pearson method. The normality of the variables was evaluated graphically and by using the Shapiro–Wilk W test. All analyses were conducted using Stata 17.0 (StataCorp, College Station, TX, USA).

## Results

Overall, 182 patients with JIA receiving ETN were eligible in the study. Two patients with inadequate compliance were excluded. Finally, 180 patients were included: 109 (61%) girls and 71 (39%) boys. The mean patient age was 8.0 years (range: 2–17 years).

The characteristics of the patients are presented in Table 1. Ninety-seven (54%) of the patients used concomitant MTX, and 83 (46%) received either ETN monotherapy or used sDMARDs other than MTX. Twenty-three patients used leflunomide, eight used sulfasalazine, and three used hydroxychloroquine (Table 2). Compared with the non-MTX group, patients in the MTX group were younger and had shorter disease duration at ETN treatment initiation. No significant difference was observed between the groups in body composition measures, disease activity, neither in the presence of ANA nor HLA-B27 antigen. CCP-ab was positive in all patients with RF-positive polyarthritis.

**Table 1:** Clinical and demographic characteristics of the patients at the time of ETN measurement

	MTX group *n* = 97	non-MTX group *n* = 83	*p* value
Female (%)	62 (64)	47 (57)	0.32
Age (years), mean (SD)	7.5 (3.6)	8.6 (3.8)	0.037
Height (cm), mean (SD)	122 (23)	128 (239)	0.11
Weight (kg), mean (SD)	26.5 (13.0)	28.6 (13.7)	0.27
BMI, kg/ m^2^	16.6 (2.8)	16.5 (2.5)	0.77
BSA (m^2^), mean (SD)	0.94 (0.31)	1.00 (0.33)	0.17
Disease duration (years), mean (SD)	2.3 (2.4)	3.2 (2.8)	0.019
Diagnosis			0.74
Oligoarthritis, persistent	17 (18)	18 (22)	
Oligoarthritis, extended	15 (15)	14 (17)	
Polyarthritis, RF-negative	57 (49)	40 (48)	
Polyarthritis, RF-positive	2 (2)	1 (1)	
Enthesitis related arthritis	4 (4)	7 (8)	
Psoriatic arthritis	1 (1)	2 (2)	
Undifferentiated arthritis	1 (1)	1 (1)	
Uveitis, n (%)	9 (9)	2 (2)	0.066
Inflammatory bowel disease, n (%)	1 (1)	1(1)	0.99
Previous bDMARD, n (%)	10 (10)	15 (18)	0.13
Etanercept	7	10	
Adalimumab	3	4	
Infliximab	2	4	
Tocilizumab	0	2	
Concomitant treatment n (%)			
Other sDMARDs	6 (6)	32 (39)	< 0.001
Prednisolone	4 (4)	7(8)	0.23
ESR (mm/h), mean (SD)	13.2 (14.0)	12.1 (12.1)	0.61
CRP (mg/l), mean (SD)	4.9 (12.6)	5.9 (13.5)	0.63
JADAS10, mean (SD)	10.0 (5.6)	9.7 (5.8)	0.56
PaGA, mean (SD)	3.3 (2.6)	2.6 (2.2)	0.10
PhGA, mean (SD)	3.1 (1.8)	2.7 (1.9)	0.14
HLA-B27 positive, n (%)	27 (28)	25 (30)	0.58
ANA, n (%)	31 (32)	23 (28)	0.54
Erosions, n (%)	21 (22)	16 (19)	0.60

*ETN* etanercept, *MTX* methotrexate, *BMI* Body mass index, *BSA* Body surface area, *RF* Rheumatoid factor, *bDMARD* biological disease-modifying antirheumatic drug, *sDMARD* synthetic disease-modifying antirheumatic drug, *ESR* Erythrocyte sedimentation rate, *CRP* C-reactive protein, *JADAS10* 10-joint Juvenile Arthritis Disease Activity Score, *PaGA* Patient’s global assessment of wellbeing measured on a linear analogue scale (VAS), *PhGA* Physician’s global assessment of wellbeing measured on a VAS scale, *HLA* Human leucocyte antigen B27, *ANA* Antinuclear antibody

**Table 2:** Other sDMARDs of the patients at the time of ETN measurement

sDMARD	MTX group *n* = 97	non-MTX group *n* = 83	*p* value
Leflunomide, n (%)	1(1)	23(28)	< 0.001
Hydroxychloroquine, n (%)	5(5)	3(4)	0.73
Sulfasalazine, n (%)	2(2)	8(10)	0.046
Azathioprine, n (%)	0(0)	1(1)	0.46
Prednisolone, n (%)	4(4)	7(8)	0.35

*sDMARD* synthetic disease-modifying antirheumatic drug, *MTX* Methotrexate

Median (Q1, Q3) time point for the measurement of ETN concentration was 12 (4, 30) months after ETN initiation. At that time point, median (range) MTX dose was 13.0 mg/m^2^ (5.5–24.2 mg/m^2^) and median ETN dose was 0.75 (0.49–1.47) mg/kg/week and median ETN concentration was 1.60 (0.40–6.30) µg/mL in the MTX group and 1.70 (0.60–4.90) µg/mL in the non-MTX group (*p* = 0.52 after adjusted ETN dose). Correlation between MTX dose and ETN concentration adjusted with ETN dose was 0.01 (95% Cl: -0.16 to 0.19).

**Fig. 1:**
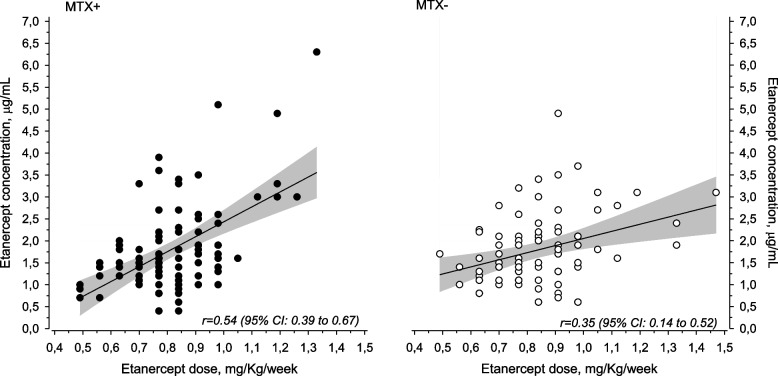
Relationship between ETN (etanercept) dose and concentration according to MTX (methotrexate) use. The grey area represents 95% confidence intervals of linear prediction

A significant correlation was revealed between ETN dose and drug level [*r* = 0.45 (95% CI: 0.33–0.56)] (Fig. 1). The ETN dose and serum drug level were correlated (*p* = 0.03) in both subgroups – in MTX group [*r* = 0.35 (95% CI: 0.14–0.52)] and in non-MTX group [*r* = 0.54 (95% CI: 0.39–0.67)]. No correlation was detected between ETN concentration and patients’ weight or body surface area.

No significant correlation was found between disease duration and ETN concentration when ETN dose was adjusted, neither in the MTX group *r* = 0.01 (95% CI: -0.15 to 0.15) nor in the non-MTX group *r* = -0.03 (95% CI: -0.23 to 0.18). Neither was significant correlation observed between disease activity and ETN concentration (Table 3).

**Table 3:** Correlations (Spearman) between ETN concentration and disease activity

	ETN concentration	
	MTX group *r* (95% CI)	non-MTX group *r* (95% CI)
ESR	-0.04 (-0.24 to 0.16)	-0.01 (-0.23 to 0.21)
CRP	-0.20 (-0.39 to -0.01)	-0.06 (-0.28 to 0.15)
PaGA	0.02 (-0.18 to 0.22)	-0.25 (-0.44 to -0.04)
PhGA	0.19 (-0.01 to 0.37)	-0.09 (-0.30 to 0.12)
JADAS10	0.13 (-0.07 to 0.32)	-0.22 (-0.42 to -0.01)

No significant correlations after Sidak adjustment

*ETN* etanercept, *MTX* methotrexate, *ESR* Erythrocyte sedimentation rate, *CRP* C-reactive protein, *PaGA* Patient’s global assessment of wellbeing measured on a linear analogue scale (VAS), *PhGA* Physician’s global assessment of wellbeing measured on a VAS scale, *JADAS10* 10-joint Juvenile Arthritis Disease Activity Score

## Discussion

To our knowledge, this is the first study to analyse ETN treatment and the effects of concomitant MTX usage on serum ETN concentration in pediatric patients with JIA receiving ETN with or without concomitant MTX. The main findings of this study are that concomitant MTX had no effect on serum ETN concentration and significant correlation was observed between ETN dose and ETN concentration. We did not observe any positive influence on clinical response in ETN-treated patients in MTX group compared with non-MTX group.

When sDMARDs are insufficient to provide remission in patients with JIA, bDMARDs are regularly used. TNF inhibitors, such as ETN, are the first choice of bDMARDs [18]. ETN has been used in JIA for over 30 years, and it has been shown to be effective and safe for long-term use [19, 20]. In a pilot study of 40 JIA patients treated with ETN, there was a clear association between circulating ETN levels, and the dose received [21], consistent with our results: increase in ETN dose was associated with increase in ETN concentration. Similarly to our study, Alcobendas et al. [21] did not find any relationship between ETN concentration and disease activity. Results of the study by Bader-Meunier et al. support these findings [22]. Also in adult patients with RA, ETN concentration did not correlate significantly with good clinial response [12].

Variation in the response to drug treatment among patients with JIA has awaken expectations to get support from therapeutic drug monitoring for decision-making during bDMARD treatment. Similar to other drugs, serum ETN concentration can be affected by several factors. ETN is administered subcutaneously, when the absorption and bioavailability is not necessarily complete. The injection site might have a minor effect on absorption accompanied by factors affecting ETN metabolism [23–25]. Moreover, it remains unclear whether body mass affects ETN concentrations, whether patients with higher body mass have higher volume on distribution [26], and whether obese patients with JIA may have difficulties in achieving remission [27]. In the present study, we did not find any correlation between ETN concentration and patients’ weight or body surface area, consistent with the results of Langley et al. [11].

ETN is a nonimmunogenic TNF inhibitor. Although antibodies are generated, they are nonneutralising and do not influence drug efficacy or safety [11, 22]. In the present study, considering the above, we did not measure anti-etanercept antibodies.

Apparently, drug concentrations in general vary widely within patients on the standard treatment dose. This intrapatient variability (IPV) is common during bDMARD treatment. Higher ETN doses might lower IPV by generating higher serum ETN concentrations and thus ensuring constant drug levels [28]. Parallel results have been reported in patients with JIA treated with ETN [29].

To our knowledge, no study has evaluated pediatric patients receiving ETN or the possible effect of concomitant MTX dosing on serum ETN concentration. In adult patients with RA receiving ETN treatment, concomitant MTX did not increase ETN concentration [12]. Deng et al. reported the influence of higher TNF-alpha concentration on ETN clearance in adult patients with ankylosing spondylitis [30], but another study revealed no association between circulating ETN concentration and concomitant MTX usage [31]. If concomitant MTX does not improve treatment outcome, it is worth of consider to taper off MTX in such patients.

In a case of a treatment failure, the problem can be that drug is ineffective and should be changed or that drug is effective, but the dose or frequency is too low. This can be determined by measuring drug concentrations. Drug trough level measurements can help in the decision of dose and frequency, and drug selection, as well as in situations where the patient is in remission, but it remains unknown whether continuing the drug administration is feasible. If the drug trough level is under the recommended level, it would be sensible to discontinue the treatment.

This study has some limitations. First, the present study was a register-based study, and clinical data were collected retrospectively from the patients’ records. On the other hand, this kind of data is valuable real-life data for clinicians. Second, considerable variation existed between the time of diagnoses of JIA and the initiation of ETN.

In conclusion, in a case of uncertainty of drug effectiveness in patients with increase disease activity, it is critical to determine whether to increase the drug dose or frequency or whether the drug is ineffective and should be altered. One possibility is to add sDMARD to the therapy if not added earlier. In the present study, we observed that MTX did not affect serum ETN concentration, but increase of the ETN dose increased its serum concentration. We found that ETN concentration did not correlate with disease activity. This might be explained by patients’ lower disease activity, when a lower ETN dose may be sufficient, or even a drug-free period. Moreover, based on the results of this study, it seems that concomitant MTX do not improve the treatment outcome. Further studies are needed to confirm our findings.

## Data Availability

The dataset used and analysed during the current study are available from the corresponding author on reasonable request.

## Abbreviations

ACR: American College of Rheumatology
ANA: Antinuclear antibody
bDMARD: Biological disease-modifying antirheumatic drug
CCP-ab: Cyclic citrulline peptide antibody
ETN: Etanercept
HLA-B27: Human leucocyte antigen B27
ILAR: International League of Associations for Rheumatology
IQR: Interquartile range
JADAS10: 10-Joint juvenile disease activity score
IPV: Intrapatient variability
JIA: Juvenile idiopathic arthritis
MTX: Methotrexate
PaGA: Patient’s global assessment of wellbeing
PhGA: Physician's global assessment of disease activity
RF: Rheumatoid factor
SD: Standard deviation
sDMARD: Synthetic disease modifying antirheumatic drug
TNF: Tumour necrosis factor
VAS: Visual analogue scale

## References

[1] Ravelli A, Martini A (2007). Juvenile Idiopathic Arthritis. The Lancet.

[2] Berntson L, Andersson-Gäre B, Fasth A, Herlin T, Kristinsson J, Lahdenne P, Nordig Study group (2003). Incidence of juvenile idiopathic arthritis in the Nordic countries. A population based study with special reference to the validity of the ILAR and EULAR criteria. J Rheumatol.

[3] Petty RE, Southwood TR, Manners P, Baum J, Glass DN, Goldenberg J (2004). International League of Associations for Rheumatology. International League of Associations for Rheumatology classification of juvenile idiopathic arthritis: second revision, Edmonton, 2001. J Rheumatol.

[4] Beukelman T, Patkar NM, Saag KG, Tolleson-Rinehart S, Cron RQ, DeWitt EM (2011). 2011 American College of Rheumatology recommendations for the treatment of juvenile idiopathic arthritis: initiation and safety monitoring of therapeutic agents for the treatment of arthritis and systemic features. Arthritis Care Res (Hoboken).

[5] Chhabra A, Oen K, Huber AM, Shiff NJ, Boire G, Benseler SM, ReACCh-Out Investigators (2020). Real-World Effectiveness of Common Treatment Strategies for Juvenile Idiopathic Arthritis: Results From a Canadian Cohort. Arthritis Care Res (Hoboken).

[6] Ringold S, Angeles-Han ST, Beukelman T, Lovell D, Cuello CA, Becker ML (2019). 2019 American College of Rheumatology/Arthritis Foundation Guideline for the Treatment of Juvenile Idiopathic Arthritis: Therapeutic Approaches for Non-Systemic Polyarthritis, Sacroiliitis, and Enthesitis. Arthritis Rheumatol.

[7] Pohjankoski H, Kautiainen H, Lauri JV, Puolakka K, Rantalaiho V (2020). Trends towards more active introduction of drug therapy, emphasizing methotrexate and biologic agents, for juvenile idiopathic arthritis. Clin Rheumatol.

[8] Moreland LW, Margolies G, Heck LW, Saway A, Blosch C, Hanna R (1996). Recombinant soluble tumor necrosis factor receptor (p80) fusion protein: toxicity and dose finding trial in refractory rheumatoid arthritis. J Rheumatol.

[9] Lovell DJ, Giannini EH, Reiff A, Cawkwell GD, Silverman ED, Nocton JJ (2000). Etanercept in children with polyarticular juvenile rheumatoid arthritis. Pediatric Rheumatology Collaborative Study Group. N Engl J Med.

[10] Yim DS, Zhou H, Buckwalter M, Nestorov I, Peck CC, Lee H (2005). Population pharmacokinetic analysis and simulation of the time-concentration profile of etanercept in pediatric patients with juvenile rheumatoid arthritis. J Clin Pharmacol.

[11] Langley RG, Kasichayanula S, Trivedi M, Aras GA, Kaliyaperumal A, Yuraszeck T (2018). Pharmacokinetics, Immunogenicity, and Efficacy of Etanercept in Pediatric Patients With Moderate to Severe Plaque Psoriasis. J Clin Pharmacol.

[12] Zhou H, Mayer PR, Wajdula J, Fatenejad S (2004). Unaltered etanercept pharmacokinetics with concurrent methotrexate in patients with rheumatoid arthritis. J Clin Pharmacol.

[13] Mosteller RD (1987). Simplified calculation of body-surface area. N Engl J Med.

[14] Kneepkens EL, Krieckaert CL, van der Kleij D, Nurmohamed MT, van der Horst-Bruinsma IE, Rispens T (2015). Lower etanercept levels are associated with high disease activity in ankylosing spondylitis patients at 24 weeks of follow-up. Ann Rheum Dis.

[15] Sanmarti R, Inciarte-Mundo J, Estrada-Alarcon P, Garcia-Manrique M, Narvaez J, Rodriguez-Moreno J (2015). Towards optimal cut-off trough levels of adalimumab and etanercept for a good therapeutic response in rheumatoid arthritis. Results of the INMUNOREMAR study. Ann Rheum Dis.

[16] Gehin JE, Syversen SW, Warren DJ, Goll GL, Sexton J, Bolstad N (2021). Serum etanercept concentrations in relation to disease activity and treatment response assessed by ultrasound, biomarkers and clinical disease activity scores: results from a prospective observational study of patients with rheumatoid arthritis. RMD Open.

[17] Griffiths CEM, Thaçi D, Gerdes S, Arenberger P, Pulka G, Kingo K (2017). EGALITY study group: a confirmatory, randomized, double-blind study comparing the efficacy, safety and immunogenicity of GP2015, a proposed etanercept biosimilar, vs. the originator product in patients with moderate-to-severe chronic plaque-type psoriasis. Br J Dermato.

[18] Onel K, Horton D, Lovell D, Shenoi S, Cuello C, Angeles-Han S (2022). 2021 American college of rheumatology guideline for the treatment of juvenile idiopathic arthritis: therapeutic approaches for oligoarthritis, tempomandibular joint arthritis, and systemic idiopathis arthritis. Arthritis Rheumatol.

[19] Swart J, Giancane G, Horneff G, Magnusson B, Hofer M, Alexeeva Ð (2018). Paediatric Rheumatology International Trials Organisation (PRINTO), BiKeR and the board of the Swedish Registry. Pharmacovigilance in juvenile idiopathic arthritis patients treated with biologic or synthetic drugs: combined data of more than 15,000 patients from Pharmachild and national registries. Arthritis Res Ther.

[20] Armaroli G, Klein A, Ganser G, Ruehlmann MJ, Dressler F, Hospach A (2020). Long-term safety and effectiveness of etanercept in JIA: an 18-year experience from the BiKeR registry. Arthritis Res Ther.

[21] Alcobendas R, Rodriguez-Vidal A, Pascual-Salcedo D, Murias S, Remesal A, Diego C (2016). Monitoring serum etanercept levels in juvenile idiopathic arthritis: a pilot study. Clin Exp Rheumatol.

[22] Bader-Meunier B, Krzysiek R, Lemelle I, Pajot C, Carbasse A, Poignant S (2019). Etanercept concentration and immunogenicity do not influence the response to Etanercept in patients with juvenile idiopathic arthritis. Semin Arthritis Rheum.

[23] Zhou H (2005). Clinical pharmacokinetics of Etanercept: a fully humanized soluble recombinant tumor necrosis factor receptor fusion protein. J Clin Pharmacol.

[24] Temrikar ZH, Suryawanshi S, Meibohm B (2020). Pharmacokinetics and Clinical Pharmacology of Monoclonal Antibodeis in Pediatric Patients. Paediatr Drugs.

[25] Verstegen RHJ, McMillian R, Feldman BM, Ito S, Laxer RM (2020). Towards therapeutic drug monitoring of TNF inhibitors for children with juvenile idiopathic arthritis: a scoping review. Rheumatology (Oxford).

[26] Giani T, De Masi S, Maccora I, Tirelli F, Simonini G, Falconi M (2019). The Influence of Overweight and Obesity on Treatment Response in Juvenile Idiopathic Arthritis. Front Pharmacol.

[27] Balevic SJ, Becker ML, Gonzalez D, Funk RS (2021). Low etanercept concentrations in children with obesity and juvenile idiopathic arthritis. J Pediatr Pharmacol Ther.

[28] Van Bezooijen JS, Schreurs MWJ, Koch BCP, Velthuis HT, van Doorn MBA, Prens EP (2017). Intrapatient Variability in the Pharmacokinetics of Etanercept Maintenance Treatment. Ther Drug Monit.

[29] Nassar-Sheikh RA, Schonenberg-Meinema D, Bergkamp SC, Bakhlakh S, de Vries A, Rispens T (2021). Therapeutic drug monitoring of anti-TNF drugs: an overview of applicability in daily clinical practice in the era of treatment with biologics in juvenile idiopathic arthritis (JIA). Pediatr Rheumatol Online J.

[30] Deng Y, Hu L, Qiang W, Cheng Z, Wang L, Wang X (2018). TNF-α level affects etanercept clearance: TNF- α concentration as a new correction factor of allometric scaling to predict individual etanercept clearances in patients with ankylosing spondylitis. Clin Exp Pharmacol Physiol.

[31] Berkhout LC, l'Ami MJ, Krieckaert CLM, Vogelzang EH, Kos D, Nurmohamed MT (2020). The effect of methotrexate on tumour necrosis factor concentrations in etanercept-treated rheumatoid arthritis patients. Rheumatology (Oxford).

